# Blunted No-Go P3 predicts faster increases in disordered eating symptoms in early adolescents: A four-wave longitudinal study

**DOI:** 10.21203/rs.3.rs-9715122/v1

**Published:** 2026-05-28

**Authors:** Jaron X. Y. Tan, Pan Liu

**Affiliations:** University of Alberta; University of Alberta

**Keywords:** inhibitory control, disordered eating, early adolescence, event-related potentials, longitudinal, multilevel growth models

## Abstract

In Canada, about 1.7 million individuals experience an eating disorder at any given time. Early adolescence is a high-risk period for the onset of disordered eating attitudes and behaviors; without timely intervention, these problems may persist into adulthood and predict a host of negative outcomes. Understanding the mechanisms associated with the development of disordered eating is critical for early risk identification and intervention. One such risk factor is inhibitory control (IC)—the ability to suppress a prepotent response to support goal-directed behavior. However, most research linking IC to eating pathology has been cross-sectional and has relied on self-report or behavioral measures of IC. This study examined to what extent the No-Go P3, an ERP correlate of IC, predicted changes in disordered eating symptoms over a period of two years during early adolescence. A group of 115 healthy early adolescents (66 girls; Mean/SD age at T1 = 10.94/1.18 years) completed an EEG Go/No-Go task at T1 and reported on their eating problems from T1 to T4 over two years. Multilevel growth models demonstrated that youths with a smaller No-Go P3 at T1, which might indicate inefficient recruitment of neural resources underlying IC, showed faster increases in disordered eating symptoms over time. No association was found for commission errors of IC. These findings suggest that neural indices of risks for eating pathology may emerge prior to overt behavioral manifestations during early adolescence, highlighting the utility of neural markers for early risk identification and prevention efforts.

## 1. Introduction

In Canada, approximately 1.7 million individuals experience an eating disorder at any given time (Galmiche et al., 2019). Early adolescence is a high-risk period for the first onset of disordered eating attitudes and behaviors, such as elevated concerns about body weight and shape, and disrupted eating behaviors that involve excessive weight control (Murray et al., 2022; Sacrato et al., 2010). Without timely intervention, these problems can persist into adulthood and are often associated with long-term physical and psychological consequences, including later eating disorders and other comorbid conditions (Neumark-Sztainer et al., 2006; O’Brien et al., 2017). Therefore, understanding the mechanisms associated with the onset and maintenance of disordered eating during early adolescence is critical for early identification of risks and the development of effective intervention for youths.

One identified risk factor for eating pathology is inhibitory control (IC), the ability to suppress a prepotent response in order to facilitate goal-directed behavior (Logan & Cowan, 1984). IC has been studied in relation to various mental health outcomes, including anxiety and depression (Thomas et al., 2022; Wright et al., 2014). Likewise, emerging studies using self-report and behavioral measures of IC have linked this construct to disordered eating symptoms across development (Bartholdy et al., 2016; Ramalho et al., 2023). However, self-report and behavioral indices may not fully capture early vulnerabilities for psychopathology, particularly during childhood and early adolescence when biomarkers of risk may emerge prior to overt symptoms or impairments (Manoach & Agam, 2013). Recent work has increasingly incorporated neural measures, such as electroencephalography/event-related potentials (EEG/ERPs), to examine the neurophysiological correlates of IC and their role in conferring risk for psychopathology, including eating pathology (Reyes et al., 2015; Thomas et al., 2024). However, most of these work has focused on adults and has relied on cross-sectional designs.

Early research has indicated that lower self-reported IC is linked to greater overeating tendencies and unhealthy eating behaviors in adults (Bartholdy et al., 2016; Ramalho et al., 2023). Compared to healthy-weight controls, obese adults reported higher impulsive tendencies (Mobbs et al., 2010). Similarly, youths aged 14 to 16 who reported greater difficulties in IC endorsed more uncontrolled eating behaviors (Nelson et al., 2020). Beyond self-report findings, behavioral studies showed that adults with bulimia nervosa exhibited longer reaction times when inhibiting their responses on a reaction task relative to healthy controls (Wu et al., 2013). Overweight adults with low dietary restraint also made more errors on a food-based response inhibition task compared to lean adults (Price et al., 2015).

In the adolescent literature, behavioral findings of IC in relation to eating pathology have remained equivocal. While some studies found significant associations between behavioral performance of IC and eating pathology in youths (Ames et al., 2014; Schaumberg et al., 2020), others did not (Nelson et al., 2020; Thomas et al., 2024; Walk et al., 2020). Among youths aged 14 to 17, higher error commissions on an IC task was associated with more binge eating behaviors in females, and greater intake of palatable food in males (Ames et al., 2014). In a longitudinal study, children with better IC at age 10 were less likely to have binge eating symptoms at age 14 (Schaumberg et al., 2020). However, another study reported no differences in response inhibition performance between 10- to 12-year-olds with and without disordered eating symptoms (Thomas et al., 2024). These mixed findings suggest that early risks associated with IC may not always emerge or be detectable at the behavioral level, particularly in youth samples.

ERP studies have further examined the neurophysiological correlates of IC in relation to eating problems (Bartholdy et al., 2019; Thomas et al., 2024). With its high temporal resolution, this technique is particularly useful in examining how neurocognitive processes like IC unfold and capturing risk processes beyond what can be detected by behavioral measures (Luck, 2014). One IC-related ERP component that has been linked to eating pathology is the inhibition-related P3, a positive deflection peaking approximately 300–600 ms following stimulus onset over frontocentral regions (Polich, 2007). In response inhibition tasks such as the Go/No-Go, *stop* signals (i.e., the cue to inhibit responses) tend to elicit a larger P3 relative to *go* signals (Wessel & Aron, 2015). In children and adolescents, a larger inhibition-related P3 tend to be associated with more successful response inhibition, indicating more efficient mobilization of neurocognitive resources in executing IC (Cragg et al., 2009). The inhibition-related P3 changes across development, with the amplitude increasing and the latency decreasing (i.e., an earlier peak) as youths age. Research also found a developmental shift in the topography of the inhibition-related P3, which gradually moves away from parietal regions and towards frontocentral regions beginning around six years of age and continuing throughout adolescence (Jonkman, 2006; Paitel & Nielson, 2021; Wessel & Aron, 2015). These changes may reflect more efficient IC as cognitive control abilities mature over time (Johnstone et al., 2007).

The inhibition-related P3 has been associated with various outcomes in adolescents, including aggression (Petersen et al., 2018; Sun et al., 2020), depression (Chen et al., 2025; Santopetro et al., 2022) and anxiety (Santopetro et al., 2022). However, fewer studies have examined its association with eating pathology, particularly during early adolescence. In adults, individuals with anorexia nervosa exhibited smaller P3 amplitudes compared to healthy controls (Yue et al., 2020). Among 9- to 10-year-old youths, those who were overweight or obese showed smaller P3 amplitudes compared to their normal-weight peers (Reyes et al., 2015; Walk et al., 2020). Another study, however, found no correlations between the P3 and disordered eating symptoms in 10- to 12-year-olds (Thomas et al., 2024). Notably, all existing studies were cross-sectional; longitudinal work is needed to examine the prospective associations between the inhibition-related P3 and changes in disordered eating during early adolescence.

Using a multilevel modelling (MLM) approach, the current study examined to what extent the inhibition-related P3 and commission error rate at baseline predicted changes in disordered eating over the course of two years in a group of community-dwelling 9- to 12-year-olds. Based on the literature on the association between P3 and eating pathology (Reyes et al., 2015; Walk et al., 2020), we expected that a smaller baseline P3, reflecting less efficient recruitment of neural resources in performing IC, would predict faster increases in disordered eating symptoms over time (i.e., a Time × P3 interaction). Similarly, based on existing behavioral findings (Ames et al., 2014; Schaumberg et al., 2020), we expected that higher commission errors of IC would predict faster increases in disordered eating symptoms over time (i.e., a Time × Error Rate interaction).

## 2. Methods

### 2. 1. Participants & Procedure

Data for the current study were drawn from a larger project investigating the cognitive risks for psychopathology. At the beginning of the study (T1), 115 community-dwelling early adolescents (66 girls, Mean/SD age = 10.94/1.18 years) and their caregivers were recruited from a Midwestern urban area in the US and invited for a lab visit. None of the youths reported lifetime or current major physical or mental illness. Sample demographics were relatively representative of the local community (87.5% White, 3.6% Asian, 8.9% More than one race; 7.2% Hispanic or Latino; family annual income: $15,000–$350,000). After obtaining caregiver consent and youth assent, youths completed four EEG tasks (including the Go/No-Go task) in a counter-balanced order and an eye-tracking task assessing different cognitive biases. After the lab visit, youths completed questionnaires on Qualtrics, reporting on their problematic eating behaviors and other psychopathological symptoms.

We collected follow-up data from the same sample approximately 6 months (T2; Mean/SD of T1–T2 interval = 6.20/1.16 months), 12 months (T3; Mean/SD of T1–T3 interval = 11.78/1.27 months) and 22 months (T4; Mean/SD of T1–T4 interval = 22.27/1.30 months) after T1. Out of the 115 youths, 95 participated at T2 (56 girls, Mean/SD age = 11.58/1.16 years), 92 participated at T3 (53 girls, Mean/SD age = 12.05/1.19 years), and 62 participated at T4 (36 girls, Mean/SD age = 12.91/1.21 years). At all follow-ups (T2–T4), youths completed the same questionnaires as T1. At T3, youths also completed the same lab tasks as T1. Participants received monetary compensation upon completing each wave. The study protocol was approved by the university’s Institutional Review Board. In this paper, only the data of the Go/No-Go task from T1 and youth self-report disordered eating behaviors from T1 to T4 were reported.

### 2. 2. Self-Report Measures

#### Child Eating Disorder Examination Questionnaire–Short (ChEDE-Q8).

Across all waves (T1–T4), youths completed the child-report version of the Eating Disorder Examination Questionnaire–Short (ChEDE-Q8; Kliem et al., 2017). The ChEDE-Q8 consists of eight items assessing disordered eating behaviors and attitudes in 7- to 18-year-olds, including restrictive eating and concerns about body weight, shape, and eating. For each item, participants selected the option that best described themselves over the past two weeks on a seven-point scale ranging from 0 (*no days*) to 6 (*every day*; e.g., “Have you felt fat?”). Item scores were summed to generate a total disordered eating score (Range = 0–48), with higher scores reflecting greater eating pathology. Internal consistency in the current sample was high (Cronbach’s α = .92 at T1, .93 at T2, .93 at T3, and .92 at T4).

#### Body Mass Index Percentile (BMI%).

Youths’ BMI was calculated using self-reported height and weight (kg/m^2^). BMI values were then converted to age- and sex-adjusted BMI% based on normative data from the Centers for Disease Control and Prevention (CDC, 2022) using the R *cdcanthro* package (Freedman, 2026). BMI% reflects each youth’s relative standing compared to their peers of the same age and sex in the United States (e.g., a BMI% of 40 indicates that a child’s BMI is higher than 40% of peers of the same age and sex). Self-reported height and weight have been shown to strongly correspond with objectively measured values in children, with meta-analytic evidence demonstrating high correlations (*r* > .80) among 6- to 11-year-olds (Rios-Leyvraz et al., 2022).

#### Children’s Depression Inventory.

Given the high comorbidity between eating pathology and depression (Bulik, 2002), we assessed youth’s depressive symptoms using the 27-item child self-report version of Children’s Depression Inventory (CDI; Kovacs, 1978). Due to limited inter-individual variability, we excluded the item ‘I want to kill myself.’ For each of the remaining 26 items, youths were asked to select one of three statements that best described them for the past two weeks (e.g., *I am sad… 0 = once in a while, 1 = many times, or 2 = all the time*). A total score was calculated to indicate depressive symptom severity (Cronbach’s α = .90 at T1, .94 at T2, .92 at T3, and .94 at T4).

### 2. 3. The EEG Go/No-Go Task

We adopted a visual Go/No-Go task in combination with EEG recordings to elicit the inhibition-related P3 (Polich, 2007; Wessel & Aron, 2015). The Go/No-Go task consisted of 252 Go trials and 63 No-Go trials divided into 3 blocks (84 Go and 21 No-Go trials per block, presented in a random order) with a self-paced break after each block. Each trial started with a fixation cross that lasted for 200 ms. Next, a black-and-white image of a dog or a cat was presented for 300 ms. Participants were instructed to press a button as quickly as possible when they saw a cat (Go stimuli) and not to press the button when they saw a dog (No-Go stimuli). The task proceeded to the next trial upon participants’ response; in the case of no response, a fixation appeared for 900 ms before moving on to the next trial. Participants took approximately six minutes to complete the task. The task was conducted using the E-Prime software (Psychology Software Tools Inc., Pittsburgh, PA).

Youths completed the Go/No-Go task in an electrically shielded chamber while continuous EEG signals were recorded using a 64-channel HydroCel Geodesic Sensor Net (Electrical Geodesic Inc.) and an EGI 200 NetAmps Amplifier. The EEG signals were recorded with a sampling rate of 250 Hz, referenced to the vertex electrode (Cz). Electrode impedances were kept below 50 kΩ. The EEG data were processed using the EEGLab (Delorme & Makeig, 2004) and ERPLab (Lopez-Calderon & Luck, 2014) toolboxes operated in MATLAB version 9.10.0 (Mathworks, Inc., Natick, MA). The raw EEG data were first filtered within the 0.1–40 Hz bandpass and re-referenced to the average of the two mastoid electrodes. Next, the data were time-locked to the onset of the Go and No-Go stimuli and segmented into epochs from 200 ms pre-onset to 1000 ms after onset, with a 200 ms pre-onset baseline correction. Finally, we rejected segments with (1) voltage beyond ±100 μV, (2) a >50 μV change of voltage between timepoints, or (3) a >300 μV change of voltage between the most positive and most negative timepoints within a 200 ms moving window. Only trials with correct responses were included. Following artifact rejection, we retained the ERP data of participants with 10 or more trials in each condition (*n* = 102; Boudewyn et al., 2018; Olvet & Hajcak, 2009).

**Figure 1:**
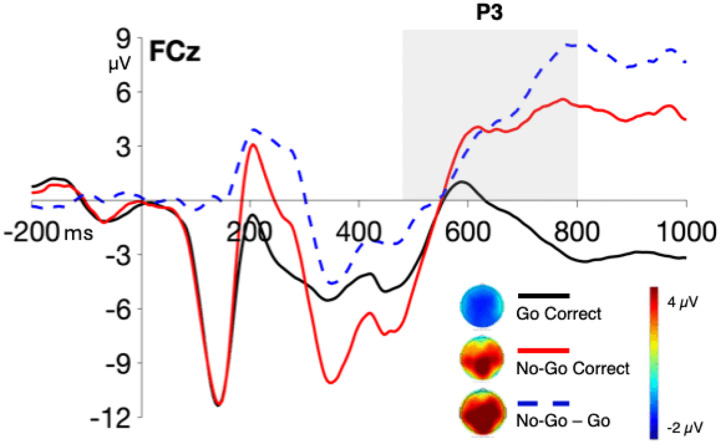
Grand average ERP waveforms and topographic maps of the P3 component at FCz for Go Correct trials, No-Go Correct trials, and the difference between the two (No-Go minus Go Correct) *Note*. μV: microvolts; ms: milliseconds.

Previous work has demonstrated a developmental shift in scalp distribution of the No-Go P3 from parietal regions to more frontocentral regions around six years of age (Jonkman, 2006; Wessel & Aron, 2015). Despite this topographic shift, a parietal P3 often remains observable, likely reflecting the engagement of more general attentional processes during task performance (e.g., stimulus differentiation; Jonkman, 2006; Jonkman et al., 2003). Consistent with this, visual inspection of the grand average waveforms in the current study showed both a frontocentral and parietal P3. Based on the literature (Bartholdy et al., 2019; Wessel & Aron, 2015), we focused on the frontocentral P3 as an indicator of IC and quantified it as the mean amplitude of the 500–800 ms time window following stimulus onset (Go and No-Go) across six frontocentral channels (F1, Fz, F2, FC1, FCz, FC2). The averaged P3 amplitude across trials in the No-Go condition was then regressed on that in the Go condition (Meyer et al., 2017) to compute standardized residual scores for each participant. The residual scores represent the unique variance of the No-Go P3 independent of general task-related activity and were used in our subsequent analyses. Figure 1 illustrates the grand average ERP waveforms across the Go Correct trials, No-Go Correct trials, and the difference between the two (No-Go minus Go) at the FCz. Paired-samples *t*-test indicated that the No-Go P3 amplitude (Mean/SD = 2.54/4.43) was significantly larger than the Go P3 amplitude, (Mean/SD = 0.96/1.80, *t*(101) = 7.30, *p* < .001).

### 2. 4. Multilevel modelling

We conducted stepwise MLM using the *lme4* package in R Studio (Bates et al. 2015), with missing data handled using full-information maximum likelihood estimation. We examined the rate of change in disordered eating across the four waves and to what extent the changes in disordered eating varied as a function of neural and behavioral indices of IC at T1: the inhibition-related P3 and commission error rate. Data were structured such that repeated waves were nested within participants. We ran two separate multilevel models with T1 residualized P3 and commission error rate as the between-person predictor, respectively. Time was treated as a within-person predictor (T1 = 0, T2 = 1, T3 = 2, and T4 = 3), with disordered eating as the outcome. Random intercepts and random slopes were included to account for individual variability in baseline levels and rates of change in disordered eating. The intraclass correlation (.54) based on an empty multilevel model indicated that approximately 54% of the variance in disordered eating over the four waves of our study was due to between-person differences.

In each model, we first entered Time as a within-person predictor of the intercept and slope of disordered eating symptoms (Step 1). We then added the between-person predictor (P3 or Error Rate) and the corresponding interaction term (Time × P3 or Time × Error Rate) in each model (Step 2). Age, Sex (0 = boy, 1 = girl), BMI% and depressive symptoms at T1 were further included as between-person covariates.

## 3. Results

Table 1 shows the descriptives and bivariate correlations of the study variables. Compared to boys, girls showed a smaller residualized P3 and made less commission errors at T1. Girls showed higher depressive symptoms at T1 and T2 and higher disordered eating symptoms at T4 relative to boys. Older age was correlated with faster RTs towards Go stimuli. Slower Go RTs were associated with less commission errors at T1 and higher depressive symptoms at T3. Disordered eating symptoms, BMI%, and depressive symptoms showed strong stability over time, with levels at each wave significantly correlated with other waves. Disordered eating symptoms were positively correlated with both BMI% and depressive symptoms across all waves. Higher depressive symptoms at T1 to T4 were associated with higher BMI% at T4. Finally, higher depressive symptoms at T2 were associated with higher BMI% at T3, and higher BMI% at T3 was associated with higher depressive symptoms at T4.

**Figure 2:**
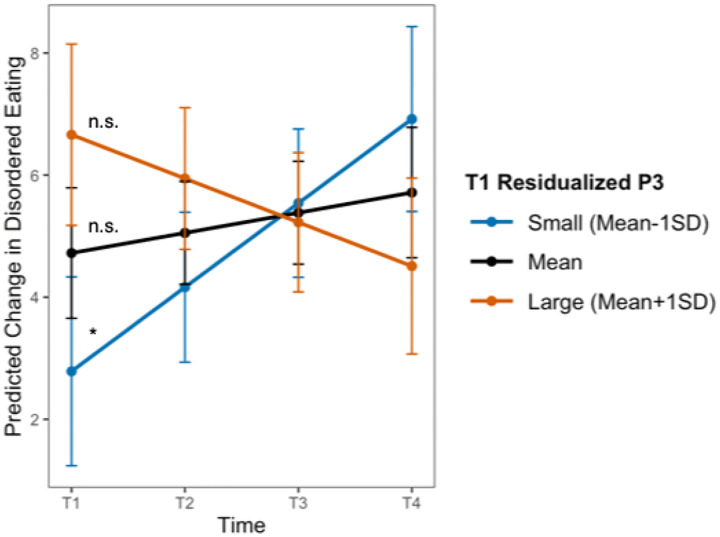
The interaction between Time and T1 residualized P3 in predicting disordered eating symptoms. *Note*. * *p* < .05; n.s.: non-significant; T1: Time 1; T2: Time 2; T3: Time 3; T4: Time 4; SD: standard error.

Table 2 and Table 3 present the MLM results. In Step 1, with Time as the within-person predictor only, we found no significant effect (γ = 0.20, *SE* = 0.48, *p* = .681); disordered eating symptoms did not change from T1 to T4 overall. In Step 2, with T1 residualized P3 as the between-person predictor (Table 2), we found a Time × P3 interaction (γ = −0.24, *SE* = 0.10, *p* = .019). Probing this two-way interaction showed that the effect of Time was significant for youths with smaller (mean-1SD; γ = 1.43, *SE* = 0.64, *p* = .027), but not mean (γ = 0.41, *SE* = 0.45, *p* = .372), or larger (mean+1SD; γ = −0.62, *SE* = 0.62, *p* =. 326) P3 (see Figure 2). Controlling for age, sex, depressive symptoms and BMI% at T1, a smaller P3 at T1 predicted faster increases in eating problems across the four waves. With T1 commission error rate as the between-person predictor (Table 3), we found neither a main effect of Error Rate nor a significant Time × Error Rate interaction (*p*s > .380).

## 4. Discussion

Despite a growing literature on the associations between IC and eating pathology, most existing work has relied on self-report and behavioral measures of IC and have primarily focused on adult populations (Bartholdy et al., 2016; Ramalho et al., 2023). A few recent cross-sectional studies have examined the link between neurophysiological indices of IC and eating pathology in adolescents (Thomas et al., 2024). To extend this work, we examined the relations between the inhibition-related P3 and changes in disordered eating symptoms in a group of community-dwelling 9- to 12-year-olds across four waves over a two-year period. Our multilevel models showed a significant Time · P3 interaction in predicting disordered eating symptoms, such that youths with a smaller, but not larger, residualized P3 at baseline reported faster increases in disordered eating symptoms over time. Contrary to our expectations, commission error rate at baseline did not predict changes in disordered eating symptoms over time (i.e., no Time · Error Rate interaction). These findings contribute important evidence to the link between neurocognitive correlates of IC and changes in disordered eating symptoms during early adolescence.

In the ERP literature, the P3 component has been linked to attentional allocation and the engagement of cognitive control processes toward task-relevant events (Polich, 2007). In response inhibition paradigms (e.g., Go/No-Go), the magnitude of the P3 has been considered an indicator of the recruitment and mobilization of neural resources in support of IC (Johnstone et al., 2007; Wessel & Aron, 2015). Altered patterns of the inhibition-related P3 have been associated with different forms of negative outcomes in children and adolescents. For example, in the context of heightened adversity such as childhood maltreatment, a larger P3 has been linked to greater externalizing behaviors (fCui et al., 2021) and anxiety symptoms (Shackman et al., 2007) in children aged 9 to 12. On the other hand, in low-risk, healthy children and adolescents, a *smaller* inhibition-related P3 has been found to be related to various psychopathology outcomes, including aggression (Sun et al., 2020), externalizing behaviors (Hosch et al., 2023; Petersen et al., 2018), and depressive symptoms (Santopetro et al., 2022). Researchers highlighted that in the context of low-risk, typical development, a smaller inhibition-related P3 may reflect inefficient mobilization of neural resources towards inhibiting dominant but goal-irrelevant responses. In our low-risk early adolescent sample, youths with a smaller P3 might have difficulties mobilizing neurocognitive resources efficiently in executing IC during the task. These individuals might similarly experience difficulties inhibiting eating-related impulses and behaviors, which, in turn, might have increased their susceptibility to eating-related symptoms over time (Appelhans et al., 2011). In contrast, youths with a larger P3 might be better able to engage in cognitive control processes when needed and inhibit goal-irrelevant impulses more effectively, reducing their risk for eating-related symptoms over time.

We did not observe an effect of baseline commission error rate of IC in predicting changes in disordered eating symptoms. While this was contrary to our expectations, it was unsurprising given that extant findings on the association between the behavioral indices of IC and disordered eating in youth have remained equivocal. Some studies found that higher commission errors in response inhibition tasks were associated with risk for obesity and more eating problems in adolescents (Ames et al., 2014; Ramalho et al., 2023). Others found no associations between behavioral performance of IC and eating problems in adolescents (Andreu et al., 2024; Bartholdy et al., 2019; Walk et al., 2020).

Previous work has suggested that the inhibition-related P3 and commission error rate may index distinct aspects of IC (Brydges et al., 2014). The P3 involves the neural processes preceding the inhibitory response, whereas the commission error rate directly reflects the success or failure of inhibitory responses and may be impacted by a wider range of cognitive and behavioral processes that contribute to the final output of task performance (Thomas et al., 2024; Xia et al., 2020). For example, variability in attention response strategies (e.g., speed-accuracy trade-offs) can influence the likelihood of successful response inhibition; individuals who tend to prioritize speed over accuracy are likely to commit more errors than those who prioritize accuracy over speed (Endrass et al., 2012; Tan & Liu, 2025; Tompson et al., 2020). In other words, greater commission errors in our youth sample might have partially reflected a tendency to respond quickly, rather than reflecting poor IC. Indeed, we found a correlation between faster RTs towards the Go stimuli and more errors during the task.

Alternatively, this pattern of results for commission errors may also be associated with age-related characteristics in the development of IC. In younger youths (8–12 years), poorer behavioral performance (e.g., more errors) on inhibition tasks may partially reflect the ongoing, normative maturation of cognitive control systems (Bunge & Wright, 2007; Somerville et al., 2011) and may not necessarily indicate elevated risks for disordered eating (Nelson et al., 2020; Thomas et al., 2024). In older adolescents (14–17 years) and adults with more developed cognitive control and regulatory capacities, overt behavioral difficulties in response inhibition may more clearly reflect problems with IC-related processes, potentially conferring greater risk for disordered eating (Ames et al., 2014; Bartholdy et al., 2016). Future research examining how the relations between behavioral and neural measures of IC and disordered eating may change during later adolescence is warranted to confirm this speculation.

The current study had several strengths. By assessing disordered eating across four time points over a two-year period, we were able to examine the directional relations between baseline IC and changes in disordered eating symptoms over time. Additionally, we used both the behavioral and ERP measures of IC, which allowed for a more comprehensive examination of different facets of IC in the context of eating pathology risks. Finally, as opposed to group comparisons (e.g., between clinical and non-clinical samples), we took a dimensional approach to examine emerging eating problems in an unselected, community-dwelling youth sample. Such an approach increases statistical power and sheds light on the processes shared by both typical and atypical development (Cicchetti & Toth, 2009).

One limitation of this study was the use of a brief, global self-report measure of eating pathology (e.g., ChEDE-Q8), which did not differentiate among specific subtypes of disordered eating, such as bulimia nervosa or binge-eating disorder (Kliem et al., 2017). While studying early eating problems in community-dwelling youths can capture the early precursors of clinically significant eating disorders, it remains unclear whether IC processes are differentially associated with distinct subtypes of eating disorders (Haynos et al., 2021). Future studies are needed to examine high-risk or clinical youths with specific subcategories of disordered eating. We also relied on youths’ self-reported height and weight to calculate youths' BMI%, which might be prone to reporting biases. Nonetheless, studies have often demonstrated high correlations between self-reported and actual assessments of height and weight (Rios-Leyvraz et al., 2022). Finally, the current findings were drawn from a primarily White youth sample from middle-class families. Future work with marginalized racial/ethnic samples and lower-income families will be important to evaluate the generalizability of these findings to other populations.

In sum, our study provided important longitudinal evidence on the prospective relations between IC and changes in disordered eating symptoms during early adolescence. Specifically, youths with a smaller inhibition-related P3 showed faster increases in disordered eating symptoms over a two-year period. This suggests that alterations in the inhibition-related P3 may confer risk for eating pathology in early adolescence. We also highlighted differential associations between behavioral and neural measures of IC and disordered eating symptoms, indicating that these measures may capture distinct facets of IC processes. Together, these findings contribute important insights into the early risk processes of eating pathology and lay the groundwork for future intervention research aimed at modifying atypical IC processes and promoting adaptive regulatory strategies in youths at risk for eating pathology.

## Figures and Tables

**Table 1:** Mean, standard deviation, and bivariate correlations of the study variables

	1	2	3	4	5	6	7	8	9	10	11	12	13	14	15	16
1. Sex																
2. Age at T1 (years)	.18															
3. T1 Residualized P3	−.22*	.06														
4. T1 Commission Error Rate	−.26**	.02	.05													
5. T1 Go Correct RT (ms)	.06	−.33**	.01	−.63**												
6. T1 ChEDE	.08	.12	.05	−.08	.03											
7. T2 ChEDE	.09	.12	.04	−.16	.09	.75**										
8. T3 ChEDE	.16	.11	−.03	−.20	.19	.56**	.70**									
9. T4 ChEDE		.10	−.07	−.14	.04	.66**	.63**	.77**								
10. T1 BMI%	−.08	−.16	−.20	.08	.08	.34**	.32**	.34**	.53**							
11. T2 BMI%	−.03	−.08	−.18	.06	−.02	.38**	.31**	.35**	.44**	.85**						
12. T3 BMI%	−.01	.01	−.03	−.05	−.05	.29*	.35**	.35**	.44**	.77**	.87**					
13. T4 BMI%	.11	−.04	−.07	−.06	.07	.46**	.45**	.40**	.47**	.78**	.76**	.88**				
14. T1 CDI	.32**	.14	.10	−.05	.10	.47**	.47**	.33**	.58**	−0.02	.01	.07	.32**			
15. T2 CDI	.25*	.05	−.02	−.14	.19	.43**	.65**	.57**	.73**	.11	.19	.30**	.41**	.78**		
16. T3 CDI	.20	.04	.14	−.04	.22*	.26*	.52**	.49**	.72**	.13	.14	.16	.42**	.69**	.80**	
17. T4 CDI	.20	.06	.00	−.02	−.03	.39**	.50**	.57**	.71**	.18	.20	.36*	.31*	.68**	.8**	.80**
Mean	-	10.94	0.00	0.42	398.60	5.77	4.97	6.24	7.17	58	61.07	56.43	57.84	8.07	7.58	9.06
SD	-	1.18	4.42	0.18	74.76	10.45	9.98	10.54	10.85	32.64	31.32	29.72	29.68	8.04	9.01	8.45

Note.

* *p* < .05,

** *p* < .01 (2-tailed);

T1: Time 1; T2: Time 2; T3: Time 3; T4: Time 4; RT: reaction time; ms: milliseconds; ChEDE: Child Eating Disorder Examination Questionnaire-Short (child version); BMI%: body mass index percentile; CDI: Children’s Depression Inventory; SD: standard deviation; Sex *(0 = boy, 1 = girl)*.

**Table 2:** Step-wise multilevel models with T1 residualized P3 predicting changes in disordered eating symptoms over time

	Estimate	SE	t	P
***Step 1***				
Intercept	5.30	1.10	4.83	< .001**
Time	0.20	0.48	0.41	.681
***Step 2***				
Intercept	1.15	2.48	0.46	.644
Time	0.41	0.44	0.93	.357
T1 Residualized P3	0.29	0.22	1.30	.197
Sex	0.49	1.48	0.33	.741
T1 Age	0.32	0.59	0.54	.592
T1 BMI%	0.10	0.02	4.54	< .001**
T1 Depressive Symptoms	0.42	0.09	4.74	< .001**
Time ´ P3	−0.24	0.10	−2.43	.019*

Note.

* *p* < .05,

** *p* < .01 (2-tailed);

T1: Time 1; BMI%: body mass index percentile; SE: standard error.

**Table 3:** Step-wise multilevel models with T1 commission error rate predicting changes in disordered eating symptoms over time

	Estimate	SE	t	P
***Step 1***				
Intercept	5.30	1.10	4.83	< .001**
Time	0.20	0.48	0.41	.681
***Step 2***				
Intercept	5.05	2.51	2.01	0.048*
Time	0.39	0.46	0.86	0.394
T1 Commission Error Rate	−4.02	5.58	−0.72	0.473
Sex	0.12	1.44	0.09	0.933
T1 Age	0.39	0.59	0.66	0.514
T1 BMI%	0.10	0.02	4.78	< .001**
T1 Depressive Symptoms	0.41	0.09	4.71	< .001**
Time ´ Error Rate	−2.44	2.76	−0.88	0.381

Note.

* *p* < .05,

** *p* < .01 (2-tailed);

T1: Time 1; BMI%: body mass index percentile; SE: standard error.

## Data Availability

Data that support the findings of this study are available from the corresponding author upon reasonable request due to due to privacy and ethical restrictions.

## References

[1] Ames S. L., Kisbu-Sakarya Y., Reynolds K. D., Boyle S., Cappelli C., Cox M. G., Dust M., Grenard J. L., Mackinnon D. P., & Stacy A. W. (2014). Inhibitory control effects in adolescent binge eating and consumption of sugar-sweetened beverages and snacks. Appetite, 81, 180–192. 10.1016/j.appet.2014.06.013 24949566 PMC4127340

[2] Andreu C. I., Cuevas A., Malbec M., Cordero M., Fuentealba J. A., & Vergés A. (2024). Diminished inhibitory control in adolescents with overweight and/or substance use: An ERP study. International Journal of Mental Health and Addiction, 22(3), 1176–1193. 10.1007/s11469-022-00922-x

[3] Appelhans B. M., Woolf K., Pagoto S. L., Schneider K. L., Whited M. C., & Liebman R. (2011). Inhibiting food reward: delay discounting, food reward sensitivity, and palatable food intake in overweight and obese women. Obesity, 19(11), 2175–2182. 10.1038/oby.2011.57 21475139 PMC3303186

[4] Bartholdy S., Dalton B., O’Daly O. G., Campbell I. C., & Schmidt U. (2016). A systematic review of the relationship between eating, weight and inhibitory control using the stop signal task. Neuroscience & Biobehavioral Reviews, 64, 35–62. 10.1016/j.neubiorev.2016.02.010 26900651

[5] Bartholdy S., O’Daly O. G., Campbell I. C., Banaschewski T., Barker G., Bokde A. L. W., Bromberg U., Büchel C., Quinlan E. B., Desrivières S., Flor H., Frouin V., Garavan H., Gowland P., Heinz A., Ittermann B., Martinot J.-L., Paillère Martinot M.-L., Nees F., … Stedman A. (2019). Neural correlates of failed inhibitory control as an early marker of disordered eating in adolescents. Biological Psychiatry, 85(11), 956–965. 10.1016/j.biopsych.2019.01.027 31122340

[6] Bates D., Maechler M., Bolker B., & Walker S. (2015). Fitting linear mixed-effects models using lme4. Journal of Statistical Software, 67(1), 1–48. 10.18637/jss.v067.i01

[7] Boudewyn M. A., Luck S. J., Farrens J. L., & Kappenman E. S. (2018). How many trials does it take to get a significant ERP effect? It depends. Psychophysiology, 55(6), e13049. 10.1111/psyp.13049 29266241 PMC5940511

[8] Brydges C. R., Fox A. M., Reid C. L., & Anderson M. (2014). Predictive validity of the N2 and P3 ERP components to executive functioning in children: A latent-variable analysis. Frontiers in Human Neuroscience, 8, 80. 10.3389/fnhum.2014.00080 24600376 PMC3929846

[9] Bulik C. M. (2002). Anxiety, depression and eating disorders. In Fairburn C.G., Brownell K.D. (Eds.), Eating disorders and obesity: A comprehensive handbook (Vol. 2, pp. 193–198). Guilford Press, NY.

[10] Bunge S. A., & Wright S. B. (2007). Neurodevelopmental changes in working memory and cognitive control. Current Opinion in Neurobiology, 17(2), 243–250. 10.1016/j.conb.2007.02.005 17321127

[11] Centers for Disease Control and Prevention. (2022). Data file for the extended CDC BMI-for-age growth charts for children and adolescents. https://www.cdc.gov/growthcharts/extended-bmi-data-files.htm

[12] Chen F., Chen C., Wu M., Luo B., Cai H., Yu F., & Wang L. (2025). Impaired emotional response inhibition among adolescents with bipolar depression: Evidence from event-related potentials and behavioral performance. BMC Psychiatry, 25(1), 303. 10.1186/s12888-025-06748-w 40165142 PMC11956177

[13] Cicchetti D., & Toth S. L. (2009). A developmental psychopathology perspective on adolescent depression. In Nolen-Hoeksema S. & Hilt L. M. (Eds.), Handbook of depression in adolescents (pp. 3–32). Taylor & Francis Group.

[14] Cragg L., Fox A., Nation K., Reid C., & Anderson M. (2009). Neural correlates of successful and partial inhibitions in children: An ERP study. Developmental Psychobiology, 51(7), 533–543. 19685486 10.1002/dev.20391

[15] Cui N., Raine A., Connolly C. A., Richmond T. S., Hanlon A. L., McDonald C. C., &

[16] Liu J. (2021). P300 Event-related potentials mediate the relationship between child physical abuse and externalizing behavior. Frontiers in Psychology, 12, 720094. 10.3389/fpsyg.2021.720094 34790145 PMC8592122

[17] Delorme A., & Makeig S. (2004). EEGLAB: An open source toolbox for analysis of single-trial EEG dynamics including independent component analysis. Journal of Neuroscience Methods, 134(1), 9–21. 10.1016/j.jneumeth.2003.10.009 15102499

[18] Endrass T., Schreiber M., & Kathmann N. (2012). Speeding up older adults: Age-effects on error processing in speed and accuracy conditions. Biological Psychology, 89(2), 426–432. 10.1016/j.biopsycho.2011.12.005 22197882

[19] Freedman D (2026). cdcanthro: Sex- and age-standardized metrics from the Centers for Disease and Control (CDC) growth charts. R package version 0.1.0, https://CRAN.R-project.org/package=cdcanthro.

[20] Haynos A. F., Wang S. B., LeMay-Russell S., Lavender J. M., Pearson C. M., Mathis K. J., Peterson C. B., & Crow S. J. (2021). An empirical taxonomy of reward response patterns in a transdiagnostic eating disorder sample. Eating Behaviors, 42, 101531. 10.1016/j.eatbeh.2021.101531 34126343 PMC8380651

[21] Hosch A., Harris J. L., Swanson B., & Petersen I. T. (2023). The P3 ERP in relation to general versus specific psychopathology in early childhood. Research on Child and Adolescent Psychopathology, 51(10), 1439–1451. 10.1007/s10802-023-01061-0 37273066 PMC10543161

[22] Galmiche M., Déchelotte P., Lambert G., & Tavolacci M. P. (2019). Prevalence of eating disorders over the 2000–2018 period: A systematic literature review. The American Journal of Clinical Nutrition, 109(5), 1402–1413. 10.1093/ajcn/nqy342 31051507

[23] Johnstone S. J., Dimoska A., Smith J. L., Barry R. J., Pleffer C. B., Chiswick D., & Clarke A. R. (2007). The development of stop-signal and Go/Nogo response inhibition in children aged 7–12 years: performance and event-related potential indices. International Journal of Psychophysiology, 63(1), 25–38. 10.1016/j.ijpsycho.2006.07.001 16919346

[24] Jonkman L. M. (2006). The development of preparation, conflict monitoring and inhibition from early childhood to young adulthood: A Go/Nogo ERP study. Brain Research, 1097(1), 181–193. 10.1016/j.brainres.2006.04.064 16729977

[25] Jonkman L. M., Lansbergen M., & Stauder J. E. A. (2003). Developmental differences in behavioral and event-related brain responses associated with response preparation and inhibition in a go/nogo task. Psychophysiology, 40(5), 752–761. 10.1111/1469-8986.00075 14696728

[26] Kliem S., Schmidt R., Vogel M., Hiemisch A., Kiess W., & Hilbert A. (2017). An 8-item short form of the Eating Disorder Examination-Questionnaire adapted for children (ChEDE-Q8). International Journal of Eating Disorders, 50(6), 679–686. 10.1002/eat.22658 28122128

[27] Kovacs M. (1978). Children's Depression Inventory (CDI) [Database record]. APA PsycTests.

[28] Logan G. D., & Cowan W. B. (1984). On the ability to inhibit thought and action: A theory of an act of control. Psychological Review, 91(3), 295–327. 10.1037/0033-295X.91.3.295 24490789

[29] Lopez-Calderon J., & Luck S. J. (2014). ERPLAB: An open-source toolbox for the analysis of event-related potentials. Frontiers in Human Neuroscience, 8, 213. 24782741 10.3389/fnhum.2014.00213 PMC3995046

[30] Luck S. J. (2014). An introduction to the event-related potential technique (2nd ed.). MIT Press.

[31] Manoach D. S., & Agam Y. (2013). Neural markers of errors as endophenotypes in neuropsychiatric disorders. Frontiers in Human Neuroscience, 7, 350. 10.3389/fnhum.2013.00350 23882201 PMC3714549

[32] Meyer A., Lerner M. D., De Los Reyes A., Laird R. D., & Hajcak G. (2017). Considering ERP difference scores as individual difference measures: Issues with subtraction and alternative approaches. Psychophysiology, 54(1), 114–122. 28000251 10.1111/psyp.12664

[33] Mobbs O., Crépin C., Thiéry C., Golay A., & Van der Linden M. (2010). Obesity and the four facets of impulsivity. Patient Education and Counseling, 79(3), 372–377. 10.1016/j.pec.2010.03.003 20399590

[34] Murray S. B., Ganson K. T., Chu J., Jann K., & Nagata J. M. (2022). The prevalence of preadolescent eating disorders in the United States. Journal of Adolescent Health, 70(5), 825–828. 10.1016/j.jadohealth.2021.11.031 35078736

[35] Nelson T. D., James T. D., Nelson J. M., Johnson A. B., Mason W. A., Yaroch A. L., & Espy K. A. (2020). Associations between specific components of executive control and eating behaviors in adolescence: A study using objective and subjective measures. Appetite, 154, 104784. 10.1016/j.appet.2020.104784 32579972 PMC7442726

[36] Neumark-Sztainer D., Wall M., Guo J., Story M., Haines J., & Eisenberg M. (2006). Obesity, disordered eating, and eating disorders in a longitudinal study of adolescents: How do dieters fare 5 years later? Journal of the American Dietetic Association, 106(4), 559–568. 10.1016/j.jada.2006.01.003 16567152

[37] O’Brien K. M., Whelan D. R., Sandler D. P., Hall J. E., & Weinberg C. R. (2017). Predictors and long-term health outcomes of eating disorders. PloS One, 12(7), e0181104. 10.1371/journal.pone.0181104 28700663 PMC5507321

[38] Olvet D. M., & Hajcak G. (2009). The stability of error-related brain activity with increasing trials. Psychophysiology, 46(5), 957–961. 10.1111/j.1469-8986.2009.00848.x 19558398

[39] Paitel E. R., & Nielson K. A. (2021). Temporal dynamics of event-related potentials during inhibitory control characterize age-related neural compensation. Symmetry, 13(12), 2323. 10.3390/sym13122323 35923222 PMC9345327

[40] Petersen I. T., Hoyniak C. P., Bates J. E., Staples A. D., & Molfese D. L. (2018). A longitudinal, within-person investigation of the association between the P3 ERP component and externalizing behavior problems in young children. Journal of Child Psychology and Psychiatry, 59(10), 1044–1051. 10.1111/jcpp.12975 30255499 PMC6467251

[41] Polich J. (2007). Updating P300: An integrative theory of P3a and P3b. Clinical Neurophysiology, 118(10), 2128–2148. 10.1016/j.clinph.2007.04.019 17573239 PMC2715154

[42] Price M., Lee M., & Higgs S. (2016). Food-specific response inhibition, dietary restraint and snack intake in lean and overweight/obese adults: A moderated-mediation model. International Journal of Obesity, 40(5), 877–882. 26592733 10.1038/ijo.2015.235 PMC4856731

[43] Ramalho S. M., Conceição E., Tavares A. C., Freitas A. L., Machado B. C., & Gonçalves S. (2023). Loss of control over eating, inhibitory control, and reward sensitivity in children and adolescents: A systematic review. Nutrients, 15(12), 2673. 10.3390/nu15122673 37375576 PMC10303700

[44] Reyes S., Peirano P., Peigneux P., Lozoff B., & Algarin C. (2015). Inhibitory control in otherwise healthy overweight 10-year-old children. International Journal of Obesity, 39(8), 1230–1235. 10.1038/ijo.2015.49 25869603 PMC4526395

[45] Rios-Leyvraz M., Ortega N., & Chiolero A. (2022). Reliability of self-reported height and weight in children: A school-based cross-sectional study and a review. Nutrients, 15(1), 75. 10.3390/nu15010075 36615731 PMC9824624

[46] Sacrato L., Pellicciari A., & Franzoni E. (2010). Emergent factors in eating disorders in childhood and preadolescence. Italian Journal of Pediatrics, 36(1), 49. 10.1186/1824-7288-36-49 20615223 PMC2912312

[47] Santopetro N. J., Kallen A. M., Threadgill A. H., Amir N., & Hajcak G. (2022). Blunted flanker P300 demonstrates specificity to depressive symptoms in females during adolescence. Research on Child and Adolescent Psychopathology, 50(4), 537–548. 10.1007/s10802-021-00876-z 34613511

[48] Schaumberg K., Brosof L. C., Lloyd E. C., Yilmaz Z., Bulik C. M., Zerwas S. C., & Micali N. (2020). Prospective associations between childhood neuropsychological profiles and adolescent eating disorders. European Eating Disorders Review, 28(2), 156–169. 10.1002/erv.2721 31994257 PMC7331088

[49] Shackman J. E., Shackman A. J., & Pollak S. D. (2007). Physical abuse amplifies attention to threat and increases anxiety in children. Emotion, 7(4), 838–852. 10.1037/1528-3542.7.4.838 18039053

[50] Somerville L. H., Hare T., & Casey B. J. (2011). Frontostriatal maturation predicts cognitive control failure to appetitive cues in adolescents. Journal of Cognitive Neuroscience, 23(9), 2123–2134. 10.1162/jocn.2010.21572 20809855 PMC3131482

[51] Sun L., Li J., Niu G., Zhang L., & Chang H. (2020). Reactive aggression affects response inhibition to angry expressions in adolescents: An event-related potential study using the emotional go/no-go paradigm. Frontiers in Psychology, 11, 558461. 10.3389/fpsyg.2020.558461 33101129 PMC7556161

[52] Tan J. X., & Liu P. (2025). Emotion regulation moderates the prospective association between ERN and anxiety in early adolescence: An age-specific moderation of cognitive reappraisal but not expressive suppression. Research on Child and Adolescent Psychopathology, 53(2), 261–277. 10.1007/s10802-024-01263-0 39585576

[53] Thomas K. S., Jones C. R., Williams M. O., & Vanderwert R. E. (2024). Associations between disordered eating, internalizing symptoms, and behavioral and neural correlates of response inhibition in preadolescence. Developmental Psychobiology, 66(3), e22477. 10.1002/dev.22477 38433461

[54] Tompson S. H., Falk E. B., O’Donnell M. B., Cascio C. N., Bayer J. B., Vettel J. M., & Bassett D. S. (2020). Response inhibition in adolescents is moderated by brain connectivity and social network structure. Social Cognitive and Affective Neuroscience, 15(8), 827–837. 10.1093/scan/nsaa109 32761131 PMC7543938

[55] Walk A. M., Raine L. B., Kramer A. F., Cohen N. J., Hillman C. H., & Khan N. A. (2020). Adiposity is related to neuroelectric indices of motor response preparation in preadolescent children. International Journal of Psychophysiology, 147, 176–183. 10.1016/j.ijpsycho.2019.10.014 31756405

[56] Wessel J. R., & Aron A. R. (2015). It's not too late: the onset of the frontocentral P3 indexes successful response inhibition in the stop-signal paradigm. Psychophysiology, 52(4), 472–480. 10.1111/psyp.12374 25348645 PMC4830357

[57] Wright L., Lipszyc J., Dupuis A., Thayapararajah S. W., & Schachar R. (2014). Response inhibition and psychopathology: A meta-analysis of go/no-go task performance. Journal of Abnormal Psychology, 123(2), 429–439. 10.1037/a0036295 24731074

[58] Wu M., Giel K. E., Skunde M., Schag K., Rudofsky G., de Zwaan M., Zipfel S., Herzog, 10.1002/eat.22143 23729277

[59] W., & Friederich H. C. (2013). Inhibitory control and decision making under risk in bulimia nervosa and binge-eating disorder. International Journal of Eating Disorders, 46(7), 721–728. 10.1002/eat.22143 23729277

[60] Xia L., Mo L., Wang J., Zhang W., & Zhang D. (2020). Trait anxiety attenuates response10.3389/fnbeh.2020.00028 PMC7078641 32218724

[61] inhibition: Evidence from an ERP study using the Go/NoGo task. Frontiers in Behavioral Neuroscience, 14, 28. 10.3389/fnbeh.2020.00028 32218724 PMC7078641

[62] Yue L., Tang Y., Kang Q., Wang Q., Wang J., & Chen J. (2020). Deficits in response inhibition on varied levels of demand load in anorexia nervosa: An event-related potentials study. Eating and Weight Disorders, 25(1), 231–240. 10.1007/s40519-018-0558-2 30168032 PMC6997249

